# Development and Validation of a New Risk-Taking Game: Helsinki Aiming Task (HAT)

**DOI:** 10.3390/bs15111597

**Published:** 2025-11-20

**Authors:** Ilmari Määttänen, Jussi Palomäki, Juha Vepsäläinen, Emilia Makkonen

**Affiliations:** 1 Department of Psychology and Logopedics, Faculty of Medicine, University of Helsinki, 00014 Helsinki, Finland; emilia.makkonen@helsinki.fi; 2 Cognitive Science, Department of Digital Humanities, Faculty of Arts, University of Helsinki, 00014 Helsinki, Finland; jussi.palomaki@helsinki.fi (J.P.); juha.vepsalainen@iki.fi (J.V.); 3 Department of Public Health and Welfare, Finnish Institute for Health and Welfare, 00300 Helsinki, Finland

**Keywords:** risk-taking, behavioral measure, self-report measure, personality, reinforcement sensitivity, HAT, BART

## Abstract

We introduce and describe a new risk-taking game, Helsinki Aiming Task (HAT), and test its construct (internal) and convergent (external) validity. HAT is a shooting game, in which the participants aim at a target under varying levels of “gun” inaccuracy and penalty for missing the target. It allows fine-grained examination of risk-taking behaviour, as it contains information on the effects of penalties and rewards on single, isolated decisions, immediately after each isolated event outcome. We validate HAT by studying individual responses to changing penalty levels and the accuracy of the “gun”, and by comparing it to behavioural and self-reported risk measures, personality traits, and socioeconomic variables. In study one (n = 51), we evaluated risk-taking responses (measured by aiming point) and their relation to other task variables (such as penalty levels and “gun” inaccuracy). In studies two to four (Ns = 66, 21, and 50), we evaluated the associations between risk-taking (measured by aiming point and accrued penalties) and sensitivity to punishment and reward (measured by shift in aiming after reward or punishment), and contrasted performance in HAT with performance in the Balloon Analogue Risk Task (BART) and self-reported risk variables. The game worked as expected: the participants became more cautious with increasing penalty levels and inaccuracy. The HAT risk-taking indicators (aiming point and accrued penalties) were weakly correlated with those of BART. HAT reward and punishment sensitivity was associated with extraversion, socioeconomic variables, and self-reported risk-taking. In combined analyses, HAT reinforcement sensitivity explained self-reported risk-taking rather well, whereas BART did not. HAT provides a new way to measure behavioural risk-taking, especially responses to positive and negative outcomes that could be interpreted as reward and punishment sensitivities.

## 1. Introduction

Risk-taking tendency, as a fundamental aspect of human behaviour, plays a pivotal role in various domains. Investigating the underlying mechanisms and influences behind individuals’ propensities to take risks not only sheds light on human cognition but also has implications for numerous real-world applications, ranging from financial decision-making to public policy formation. The exploration of why some individuals exhibit a preference for embracing uncertainty and pursuing risky outcomes while others favour more cautious approaches has garnered considerable research attention.

Risk-taking tendency has several real-life consequences: it influences health behaviour and health in general (Adler et al., 1992), substance use (Wagner, 2001), gambling behaviour (Powell et al., 1999), and propensity to injury (Turner et al., 2004) or accidents (Jonah, 1986). Individuals seem to be sensitive to environmental influences for their risk-taking. Higher economic inequality seems to increase risk-taking on both societal (Pleskac & Hertwig, 2014) and individual (Mishra et al., 2015) levels.

Humans and non-human animals are generally risk-averse, preferring low-variance options over high-variance ones (Caraco et al., 1980; Kahneman & Tversky, 1984). Individual differences in risk-taking can be studied using several different methods, including self-reported questionnaires (Zhang et al., 2018) and behavioural risk-taking tasks (Lejuez et al., 2002). Typically, the correlations between behavioural tasks and self-reported measures are not high, which prompts the question whether they are measuring the same phenomenon, that is, an underlying risk-taking tendency trait (Frey et al., 2017; Pedroni et al., 2017). The analysis by Frey et al. (2017) further suggests that self-report measures tapping propensity (e.g., questions such as “Are you generally a risk-taking person or do you try to avoid risks?”) and frequency (“How many cigarettes do you smoke per day?”) share significant variance with one another but not with behavioural measures of risk-taking. These findings highlight the possibility that everyday risk-taking, thrill seeking, and substance use are related to underlying behavioural traits that are not captured by the existing behavioural risk tasks. Risk-taking is a multifaceted and nuanced phenomenon, and the current tools used to study it leave much room for improvement.

Addressing these gaps in our understanding of risk-taking propensity is essential for advancing our knowledge in this field. By refining measurement techniques and exploring the underlying behavioural traits associated with risk-taking, we can gain deeper insights into decision-making processes and develop more accurate models for predicting and managing risk in diverse domains.

### 1.1. Existing Risk-Taking Measures: The Balloon Analogue Risk Task (BART)

The Balloon Analogue Risk Task (BART) is among the most well-known and used behavioural tasks measuring risk-taking. (Lejuez et al., 2002) In BART, participants engage in a sequential pumping activity where they virtually inflate a balloon. They have the choice to either continue pumping and accumulate more points, with larger balloons corresponding to higher point values, or stop pumping and collect the accrued points before the balloon bursts. As such, BART has been called a *sequential* or a *dynamic* risk-taking task (Pleskac, 2008; Figner et al., 2009), resembling its predecessor, Devil’s Task (originally described in Slovic, 1966, though not yet called such), and follower, Angling Risk Task (Pleskac, 2008), as well as the Columbia Card Task (Figner et al., 2009).

The objective of BART is to collect hypothetical money by filling balloons with different capacities by pumping them one pump at a time. One pump accumulates money by X amount, but all of the money collected from a particular balloon is lost if the balloon breaks. The participant pumps the balloon by pressing the spacebar and collects the money, and moves to the next balloon by pressing the enter key.

BART has an optimal number of pumps that yields the highest expected value of reward, thus success in the game is confounded with risk-taking propensity. When playing BART, participants are typically risk-averse, which leads to a lower-than-optimal expected value of rewards. Pleskac (2008) showed that one disadvantage of such sequential risk-taking tasks is the possible bias in their risk-propensity indicators, since the intended number of blows for balloons that pop is unknown, and because participants do not know the optimal number of blows in terms of expected value of rewards. In other words, for balloons that pop, participants’ intended level of risk-taking is likely higher than what they had the chance to show. Consequently, BART risk-taking indices are necessarily calculated only from balls that did not pop, which yields biased estimates of risk-propensity.

Regarding the associations between self-reported risk-taking and performance in BART, prior research has yielded mixed findings. Some studies have reported significant associations between BART performance and various psychological variables, including self-reported risk-taking behaviours (Lejuez et al., 2002). However, it is worth noting that not all studies have observed these associations (Brailovskaia et al., 2018). The correlations between self-reported risk-taking behaviour and BART performance were low (Szrek et al., 2012). This suggests that the relationship between self-reported risk-taking and performance in the BART is generally weak. These modest correlations imply that self-reported risk-taking measures may not fully capture the nuanced decision-making processes involved in the dynamic risk-taking environment of the BART. Sambol et al. (2023) further found that BART was not related to other commonly used risk tasks, such as the Columbia Card Task and the Iowa Gambling Task. They view that CCT and IGT measure risk-taking behaviour, while BART measures decision-making under uncertainty, with the level of risk unknown.

De Groot (2020) discusses the shortcomings of BART in their review. One notable disadvantage of the task is the cumulation of risk while reward stays the same: each pump makes the balloon bursting more probable, but the reward for each pump stays the same. This lowers the expected value of the later pumps in the trial: it is a certainty that the balloon will explode if the participant continues pumping. Different versions of BART have been developed to address outstanding questions (Young & McCoy, 2019), such as the recent virtual reality version by Ju and Wallraven (2023). They found a connection between BART, self-reported sensation seeking, and risky behaviour in a virtual reality driving task, but did not examine associations with self-reported risk-taking.

Generally, high correlations can be expected between self-report risk-taking scales measuring individuals’ propensity and frequency of risky attitudes and behaviours, and thus risk preferences have psychometric properties similar to psychological traits such as personality or intelligence (Frey et al., 2017). However, as argued by Frey et al. (2017), there are notable differences between stated (self-report-based) and revealed (objectively observed) risk preferences and behaviour. This raises the question of which types of tools researchers should then use to measure risk-taking, as no cross-disciplinary gold standard tool exists.

### 1.2. A New Risk-Taking Measure: Helsinki Aiming Task (HAT)

Helsinki Aiming Task (HAT) was inspired by a dart-throwing task developed by Näätänen and Summala (1975), and it differs from most other behavioural risk-taking measures, such as BART, in that it allows the inspection of immediate and gradual reactions after punishment and reward.

In Study 1, we describe the development of HAT and assess it as a behavioural risk-taking propensity measure for laboratory use. In HAT, as in BART, the risk level of each decision is learned based on feedback from earlier decisions. On the one hand, for a task to serve well as a risk-taking measure, the feedback must be as illustrative as possible and, in principle, comprehensive enough to make it possible to find the optimum strategy based on it. On the other hand, the task must be difficult enough to produce measurable differences between participants. The negative outcome must be rare enough to be felt like an exception and to evoke an emotional reaction (a minor “shock”). Risk-taking should also not be confounded with motor skill, as it was in the previous dart-throwing task in which a real dartboard was used (Näätänen & Summala, 1975).

In the dart-throwing task (Näätänen & Summala, 1975), the risk level is manipulated by altering the distance of the participant from the dartboard. The participant will obtain positive points if they are not unlucky enough to throw their dart into a sector that produces zero, and in extreme cases, negative points. HAT is implemented as a computer game, and thus the act of throwing has been replaced with the motorically simpler act of “shooting” using a mouse cursor. As with the original task, there is no time pressure. Physically throwing something is a complex task requiring many different skills, including hand–eye coordination. HAT retains the aiming metaphor and inherent inaccuracy from its predecessor. Inherent differences in accuracy between participants (i.e., differences in skill) are eliminated, however, by replacing the motor action of throwing with generated randomness, as the deviation between the aim and the hit is determined by sampling from a normal distribution. Thus, there is no completely riskless option available, and even the most cautious choice may lead to an “accident”. To limit the variation in the task duration, each participant shoots the same number of shots. In HAT, there is a left-to-right gradient in rewards and risks: in the left corner of the table, the most likely score is 1, and in the extreme end on the right, the score is 10, after which there is a line beyond which the participant will receive negative points, i.e., penalty or “punishment”. The participant has to decide, based on their risk level preference, how close to the penalty area they will aim, as the “gun” has some variation, and the “shot” does not always land in the location it was aimed at. Further details on the game are provided later, in the Section 2: Methods.

In addition to the typical risk-taking variables, i.e., aiming point, scored points, and number of penalties, HAT also makes it possible to evaluate very high-resolution decision-making for each shot. Thus, immediate changes in the shots can also be measured. In this study, we utilise this feature of the HAT risk-taking game: we evaluate the aiming change after each reward (positive score) and punishment (negative penalty score). According to Gray’s (1982) Reinforcement Sensitivity Theory, personality can be described in terms of lower-level individual differences in reinforcement sensitivity. It posits that reinforcement sensitivity may be an underlying reason for many of the personality trait differences between individuals. We argue that aiming shifts after positive points or reward and negative points or penalty or punishment may reflect reward and punishment sensitivities, and together they could tentatively be called ‘reinforcement sensitivity’, although it reflects only the first reaction to reward or punishment of the reward sensitivity—the immediate reactivity after the reward or punishment rather than memory formation.

Besides immediate and/or low-level reactivity, such as sensitivity to reward and punishment, other hypothetically relevant connections, including higher cognition, are also possible. Dopamine neurons in the midbrain take part in coding the difference between expected and acquired reward to facilitate reinforcement learning (Schultz et al., 1997). The brain also engages in this process during risk-taking (Cohen, 2007), and dopamine signalling is linked to adaptive decision-making under uncertainty (França & Pompeia, 2023). Individual differences in this process might explain risk-taking behaviours: Padmanabhan et al. (2011) found that adolescents showed increased activity in striatal regions in response to rewards, which was associated with better performance compared to non-rewarding trials. This effect was not found in adults, and the writers speculate that this heightened response may contribute to reward-biased behaviour and risk-taking.

In addition to this attention-independent subcortical reward processing, there is evidence indicating that responses in the anterior insula reflect conscious evaluation of rewards and depend on attentional resources (Rothkirch et al., 2014).

### 1.3. Convergent Validity: Associations Between HAT and Psychologically Relevant Variables

To determine the convergent validity of HAT, we sought to evaluate the associations between HAT- and BART-derived risk-taking variables. In addition, we focused on self-reported risk-taking behaviour and well-being, as measured by substance use, various psychological traits, and mental health.

These measures included socioeconomic variables (economic situation of the family for teenagers, self-reported economic worry for adults) and self-reported risky behaviour (substance use, gambling, etc.). In addition, associations between HAT and psychological well-being (depression), personality (short five (Konstabel et al., 2012) and HEXACO (de Vries, 2013)) questionnaires, and a questionnaire for self-rumination and self-reflection (Elliott & Coker, 2008). Also, self-reported preference for risky behaviour questionnaires (Grips questionnaire (Zhang et al., 2018), SIRI questionnaire (Zaleskiewicz, 2001)) were studied. The selection of these questionnaires was based on their theoretical or practical relevance to our investigation from questionnaires that are related to either risk-taking or to real consequences, such as well-being and stress.

Risk-taking is associated with mental health, well-being, and socioeconomic status (Jia et al., 2021), especially among young males. Thus, our investigation also involved examining whether the combination of socioeconomic status and variables derived from HAT performance could predict risky behaviour among adolescent males. Finally, a novel self-report measure assessing beneficial and harmful fortitude, that is, the Finnish concept of “sisu”, was also utilised, as it has been found to be highly correlated with multiple stress- and well-being related variables (Henttonen et al., 2022).

Study 2 was set up to validate HAT among the most risk-prone population: young adolescent males (Armstrong & Forste, 2013; Reniers et al., 2016). As socioeconomic status (SES) has been found to be relevant for risk-taking tendency, variables that are related to SES were included (Brieant et al., 2020).

### 1.4. Research Questions

In this study, we examine the risk-taking behaviour measured by the Helsinki Aiming Task (HAT) and its associations with various variables. We operationalise HAT risk-taking behaviour in two aspects: (1) straightforward risk-taking, including aiming location, number of penalties, and the sum score, and (2) reinforcement-sensitivity, focusing on the negative aiming shift after punishment (i.e., penalty by negative points) and positive aiming shift after reward (i.e., positive points).

Our research questions are as follows:

1. How do participants adjust their risk-taking behaviour, specifically their aiming point, in response to the risk level (amount of penalty presented at the current table) and uncertainty (inaccuracy of the “gun”)? (Study 1)
2 and 3. What are the associations between HAT risk-taking variables (aiming point, number of penalty points; RQ2) and HAT reinforcement sensitivity variables (aiming shift after reward and penalty; RQ3) the following factors:
  (a) Socioeconomic status (Study 2) and self-reported economic difficulties (Study 4)
  (b) Self-reported risk behaviour (Study 2) and self-reported risk-taking tendency (Study 4)
  (c) Personality traits (Studies 2–4)
  (d) Depressive symptoms (Study 3; only RQ2)

By addressing these research questions, we aim to gain a deeper understanding of the relationship between HAT risk-taking variables, including reinforcement sensitivity, and a range of factors such as socioeconomic status, self-reported behaviours, and personality traits.

## 2. General Methods

### 2.1. Studies 1–4: General Description of HAT

**Figure 1:**
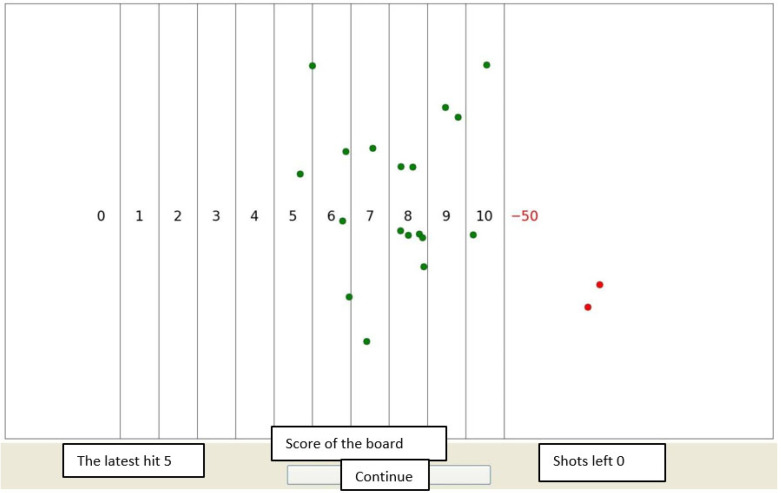
Example of a HAT board. Dots represent the shots, which are the result of the aiming point of the player and the accuracy of the gun (standard deviation from the aiming point). Green dots represent the shots that lead to obtaining positive points. Points are collected according to the shots: Each column shows the amount of points (1–10, −50). The number of points increases from left to right until the far right, which is the penalty zone. The red dots represent the shots that landed in the penalty zone. In the current table, the penalty was −50. The table has the maximum number (20) of shots. (Note that there are Finnish labels underneath the English translations).

In HAT, the subject is presented with shooting boards one at a time. The subject must decide how near to the right end (the penalty area) he or she will aim. Positive points from 1 to 10 are given, depending on how close the shot ends up to the penalty area (see Figure 1). The penalty may vary: typically, it is −50 or −100, but it may be adjusted to different scores if needed. Also, the “inaccuracy” of the aim may vary: in studies 2 and 3, the standard deviation around the aiming point of the shot was 1.5, 1.8, or 2.1; in study 4, the standard deviation from the aiming point was 1.5. After 20 shots, the subject sees their overall scores from all the boards and is presented with a new board (See Figure 1). In studies 2–4, 30 boards were used altogether. Thus, in total, each subject “shoots” 600 shots, i.e., makes 600 decisions about the risk level they decide to choose next. Naturally, most of the shots end up giving positive points, and penalty scores are relatively uncommon. The subjects avoid receiving penalties by aiming towards the left, that is, the non-penalty end of the table. HAT provides the researchers with multiple variables that can be used to study risk-taking tendencies. HAT provides points or penalties given from the tasks, the average aiming point, and the deviation from the optimal aiming point. In addition, HAT also provides information about the shift in the aim after penalties or when there is no penalty. Further research may provide more information on which of the variables are the best predictors of risk-taking preference and behaviour.

**Figure 2:**
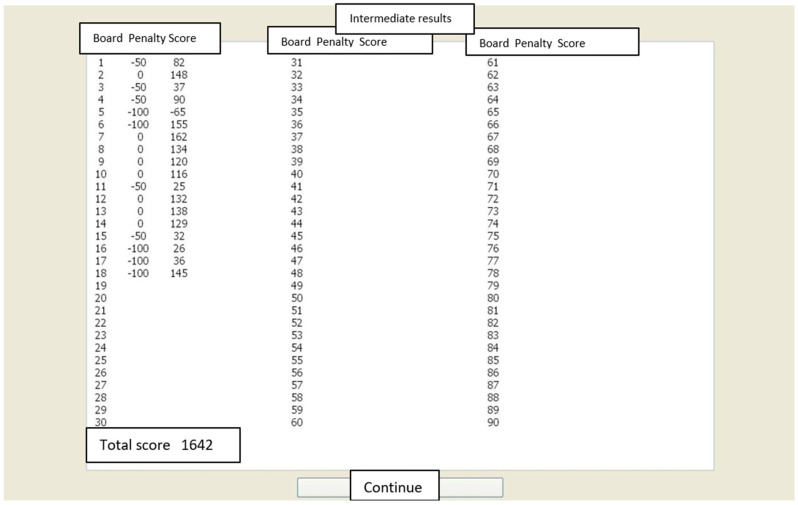
After each board, the participant saw this statistics screen of the previous shots and the upcoming new boards. In the figure, there will be 90 boards total, of which the participant has completed 18. The next board will be board number 19. (Note that there are Finnish labels underneath the English translations).

The task itself is described in Figure 1 and Figure 2. Figure 1 presents the board, into which the subjects shoot the “gun”. Figure 2 presents the intermediate results table, which appears every 20 shots. From the intermediate results table, the subject can follow their total score.

### 2.2. Studies 1–4: Statistical Analyses

The statistical analyses were performed with SPSS version 30.0.0.0 (172) or earlier. The analyses included the use of Pearson correlation and stepwise regression analyses.

Most of the questionnaire variables were from pre-existing questionnaires. In some cases (Study 2: SES, risky behaviour), variables were formed by using separate items by the authors and then summing them together to form a single variable.

Six variables indexing risk-taking propensity were calculated from HAT performance scores. The HAT *points* variable was calculated by summing up all the “winnings” and subsequently subtracting penalty scores. The *HAT aim from optimum* was calculated as the difference between the participant’s mean aiming point and the mathematical optimum aiming location. The *HAT penalty sum* was calculated by adding together all the penalties in the HAT game. *HAT penalty count* refers to how many times the participant received a penalty. The *HAT aiming shift after penalty* was calculated by adding together all the aiming shifts of the same participant. Typically, or almost always, individuals react to penalties by varying degrees of shift in the next aiming point to a “safer” direction. This could be understood and will be referred to as *punishment sensitivity* or *HAT aiming shift after the penalty* of the individual. On the other hand, the tendency to shift the aiming point to the right after each consecutive positive score was referred to as *reward sensitivity* or *HAT aiming shift after reward*. This could be understood and referred to as sensitivity to reward of the individual, because the individual is (typically very slightly) encouraged to take more risk after each positive outcome. Negative value in the variable “aiming shift after penalty” (i.e., punishment sensitivity) means that the individual has a stronger aiming shift towards lower risk and thus lower score after a penalty. The opposite is true for “aiming shift after reward” (i.e., reward sensitivity): the higher the (positive) value, the higher the aiming shift is after a shot towards the higher risk and thus higher score.

As there were no consistent associations between age, sex, and HAT risk-taking variables, age and sex were not included in the analyses.

## 3. Study 1: Initial Development and Testing of HAT

### 3.1. Study 1: Participants

Participants were recruited from the University of Helsinki’s cognitive science and related courses. In total, 50 out of the 51 participants were students, with 24 of them male (47%) and 27 (53%) female. The participants were 18–45 years old (11 did not report their age). The average age was 25.1 years (SD = 6.4).

### 3.2. Study 1: Procedure

To have validity as a measure of risk-taking propensity, HAT should not only include objective risk, but also subjective risk that influences the participants’ behaviour. Therefore, in study 1, we evaluate whether changes in probability and magnitude of the unwanted consequence (the penalty) affect the participants’ aiming. Thus, the probability of the penalty is manipulated by adjusting the accuracy of the gun in the middle of the experiment. This is achieved by changing the standard deviation parameter of the normal distribution from which the deviation between the aim and hit is picked, after the participant has finished 50% of the separate tasks with a standard deviation of 1.8. After that, 50% were randomly assigned to the low standard deviation (1.5) group and 50% were assigned to the high standard deviation (2.1) group. Also, the penalty magnitude was changing between the different tables, as the point loss resulting from a hit into the penalty zone.

The changes in aiming caused by the manipulations may result from conscious or subconscious processes. For this purpose, the participants were not informed about changes in gun accuracy, which are possibly small enough to remain subliminal (below the threshold of consciousness). The appropriate manipulation magnitudes were defined during pilot experiments before the actual trials.

As the participant entered the laboratory, they were instructed to sit in front of a computer screen and sign an informed consent form. The participant was then presented with the instructions to the task and told to start when ready. The researcher stayed in the room to observe the task for one “board” (20 shots) in case of difficulties, and then left the participant to complete the task by themselves. After the task, the participant answered to basic background information questionnaire as well as additional questions about their experiences in the whole experiment.

**Figure 3:**
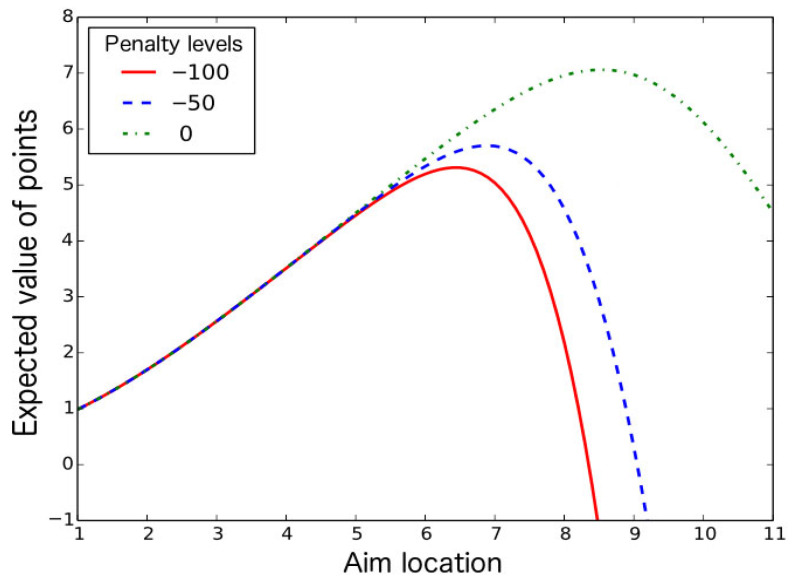
Theoretical calculation of the optimum aiming location, which depends on the penalty level of the table.

Figure 3 presents the expected number of points based on the aiming point and level of penalty. This is not visible to the participants. Table 1 presents the optimal aiming points based on different penalties and deviations. The higher the penalty and deviation are, the lower the optimal aiming point is.

Participants (N = 51) shot at 90 boards. Each target had a varying “gun accuracy” and level of penalty. Gun accuracy refers to random horizontal dispersion (standard deviation was either 1.5, 1.8, or 2.1) around the coordinates where participants aimed, and the level of penalty was the amount of penalty for hitting the penalty zone (either −100, −50, or 0). Participants were randomly assigned to either group 1 (n = 26) or group 2 (n = 25). Targets 1–45 had a gun accuracy (random horizontal dispersion) of 1.8 for both groups; targets 46–90 had a gun accuracy of either 1.5 (group 1 with smaller deviation) or 2.1 (group 2 with larger deviation). Participants’ mean aim locations and standard deviations are described in Table 2. Additional variable descriptives are presented in File S2: Supplementary Tables S1–S3 about studies 2–4.

### 3.3. Study 1 Results

**Figure 4:**
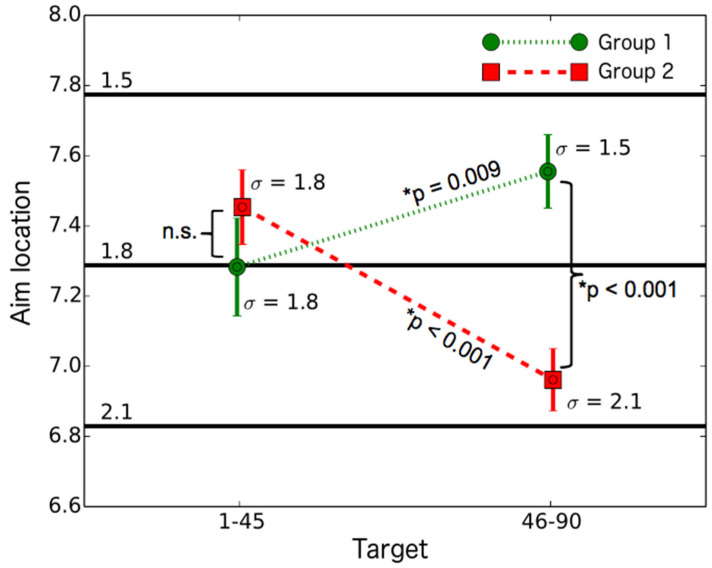
Mean aim locations across penalty levels and their changes with varying horizontal dispersion from 1.8 (targets 1–45) to 1.5 (targets 46–90 for group 1) or 2.1 (targets 46–90 for group 2) standard deviations. Horizontal lines depict optimal aim locations across penalty levels, separately for three horizontal dispersions (1.5, 1.8, and 2.1). Error bars are +/−1 standard error. * refers to statistically significant difference; n.s. refers to not (statistically) significant.

There were no significant differences in mean aim locations between groups 1 and 2 for targets 1–45 (horizontal dispersion: 1.8; U = 288, *p* = 0.486, r = 0.10; see Figure 4), suggesting that group assignment was random. However, for targets 46–90, mean aim locations differed between the groups: group 1 (horizontal dispersion: 1.5) and group 2 (horizontal dispersion: 2.1). This supports Hypothesis 1 (H1), which posits that participants with better gun accuracy would aim closer to the penalty zone (U = 115, *p* < 0.001, r = 0.55).

Within group 1, the mean aim location was closer to the penalty zone for targets 46–90 (dispersion: 1.5) compared to targets 1–45 (dispersion: 1.8), further supporting H1: as gun accuracy increased, participants aimed closer to the penalty zone (Z = 2.63, *p* = 0.009, r = 0.52).

In group 2, the mean aim location was farther from the penalty zone for targets 46–90 (dispersion: 2.1) compared to targets 1–45 (dispersion: 1.8), again supporting H1: as gun accuracy decreased, participants aimed farther away from the penalty zone (Z = 3.73, *p* < 0.001, r = 0.75).

In the combined group (horizontal dispersion, i.e., standard deviation: 1.8, targets 1–45), mean aim locations differed significantly between penalty levels. For the “−100” penalty level, mean aim locations were lower than for the “−50” level (Z = 5.63, *p* < 0.001, r = 0.79). Additionally, for the “−50” penalty level, mean aim locations were lower than for the “0” level (Z = 6.2, *p* < 0.001, r = 0.87), supporting the assumption that penalty level influences aim location.

The mean aim location in the combined group did not differ significantly from the theoretical optimal aim location (Z = 1.12, *p* = 0.265, r = 0.16), with a mean deviation of 0.079 from the optimal aim point. Of the participants, 30 aimed above the optimal location, while 21 aimed below it.

## 4. Study 2: Validation of HAT: Young Males

### 4.1. Study 2: Participants

Participants for the study were recruited from two upper comprehensive schools in Helsinki. Recruitment was facilitated by the schools’ student counsellors. A total of 66 participants took part in the study, all of whom were male. Their ages ranged from 15 to 16 years, with an average age of 15.2 years.

### 4.2. Study 2 Procedure

The participants were first given a brief explanation of the study’s purpose and procedure. They then completed an informed consent form before playing the HAT game.

After playing the game, participants completed three questionnaires: Background Information Questionnaire—included questions about risky behaviour. Short Five Personality Questionnaire—based on Konstabel et al. (2012). Consideration of Future Consequences Questionnaire (CFC)—developed by Strathman et al. (1994). For practical reasons due to issues with the experiment, two participants completed the questionnaires first and played the HAT game afterwards.

As stated, all the participants and their legal guardians gave their informed consent. In addition, permission for the study was obtained from the rector of the school.

### 4.3. Study 2 Results

In Study 2, we identified several associations between performance in the HAT game and self-reported personality traits, socioeconomic factors, and other variables. The full correlation tables are provided in Table 3.

Participants with higher levels of extraversion took more risks in the HAT game, as indicated by a higher penalty count (r = 0.293, *p* < 0.05). There was also a non-significant trending correlation between extraversion and a riskier aiming point (r = 0.261, *p* < 0.1) and with a higher penalty sum (r = 0.262, *p* < 0.1). Openness, on the other hand, was negatively correlated with penalty sum (r = −0.277, *p* < 0.05).

Socioeconomic status (log SES) was inversely associated with punishment sensitivity (indicated by aiming shifts after penalties, r = 0.361, *p* < 0.05) and reward sensitivity (indicated by aiming shifts after rewards, r = −0.348, *p* < 0.05). This suggests that participants from lower-SES families exhibited greater punishment and reward sensitivities. However, family SES did not correlate with self-reported risk-taking or substance use behaviour.

Interestingly, scores on the Consideration of Future Consequences (CFCs) scale did not correlate with any HAT game variables. However, CFC scores were positively correlated with the personality traits of conscientiousness (r = 0.321, *p* < 0.05) and openness (r = 0.474, *p* < 0.001). Additionally, CFC scores were slightly negatively correlated with self-reported risk-taking and substance use behaviour (r = −0.259, *p* < 0.05).

## 5. Study 3: Validation of HAT: Mental Health

### 5.1. Study 3: Participants

In the first stage of the study, participants were recruited through online discussion forums to complete an internet-based questionnaire.

In the second stage, participants who had responded to the online questionnaire were invited via email to participate in a laboratory session. A total of 21 participants took part, including 4 males (19%) and 17 females (81%). The participants’ ages ranged from 15 to 49 years, with a mean age of 26.7 years.

### 5.2. Study 3 Procedure

In this study, participants completed a memory task featuring both neutral and negative words prior to playing the HAT game. The analysis of the memory task is not included in this article. Participants were informed that the best-performing player—defined as the one with the highest HAT game score by the end of the experiment—would receive an additional movie ticket as a reward.

The study utilised several questionnaires that included the Short Five Personality Questionnaire (Konstabel et al., 2012), the Self-Rumination and Self-Reflection Questionnaire (Elliott & Coker, 2008), and Beck’s Depression Inventory-II (Beck et al., 1996).

Informed consent was obtained from all participants prior to their participation.

### 5.3. Study 3 Results

Study 3 confirmed the positive association between extraversion and risk-taking observed in Study 2. Specifically, extraversion was positively associated with penalty sum (r = 0.494, *p* < 0.05). Additionally, agreeableness was linked to punishment sensitivity (r = −0.706, *p* < 0.05), with more agreeable individuals showing a stronger negative aiming shift following a penalty. No significant associations were found between rumination or self-reflection and any HAT variables. However, there was a negative correlation between depressive symptoms (log BDI) and HAT points (r = −0.515, *p* < 0.05), indicating that higher levels of depressive symptoms were associated with lower HAT scores.

## 6. Study 4: Validation of HAT: Other Behavioural and Self-Reported Risk-Taking Measures

### 6.1. Study 4: Participants

Participants were primarily recruited from the University of Helsinki’s central library, where a research assistant displayed a visible information poster to attract interest. A movie ticket was offered as an incentive for participation. No exclusion criteria were applied at this stage.

A total of 50 participants took part in the study, with 28 (56%) being full-time students. The group was evenly split by gender, comprising 25 males (50%) and 25 females (50%). The participants’ ages ranged from 22 to 64 years, with an average age of 33.1 years.

### 6.2. Study 4: Procedure

The study began with participants providing informed consent. During the instructions, they were informed that the top three performers would receive an additional movie ticket, an incentive designed to encourage serious and active engagement with the tasks.

The order of the games was randomised: half of the participants began with the Balloon Analogue Risk Task (BART), while the other half started with the Helsinki Aiming Task (HAT). Participants were informed that the study aimed to explore their playing styles in relation to their questionnaire responses.

The games were played individually in a separate room. Before each game, detailed instructions were displayed on a computer screen, after which the researcher ensured the participant understood the game objectives. Periodically, the researcher briefly checked on participants to monitor their progress.

After completing both games, participants were instructed to complete an online questionnaire. This questionnaire included items about socioeconomic status, health, and well-being, as well as several validated scales: Mental Toughness Questionnaire (18 items)—Clough et al. (2002), Sisu Questionnaire (32 items)—Finnish perseverance scale by Henttonen et al. (2022), HEXACO Personality Inventory (24 items)—de Vries (2013), General Risk Propensity Scale (eight items)—Zhang et al. (2018), Stimulating-Instrumental Risk Inventory (17 items)—Zaleskiewicz (2001).

### 6.3. Study 4 Results

Study 4 revealed several associations between risk-taking behaviour, as measured by the General Risk Propensity Scale (GRiPS), and variables from the Helsinki Aiming Task (HAT). GRiPS scores were negatively correlated with HAT points (r = −0.319, *p* < 0.05) and positively correlated with HAT penalty sum (r = 0.301, *p* < 0.05). GRiPS scores were also associated with HAT punishment sensitivity (i.e., aiming shift after penalties; r = −0.348, *p* < 0.05) and HAT reward sensitivity (i.e., aiming shift toward higher scores after rewards; r = 0.397, *p* < 0.01).

Self-reported *beneficial sisu* (a Finnish concept of perseverance) was positively linked to reinforcement sensitivity in HAT, including both punishment sensitivity (r = −0.339, *p* < 0.05) and reward sensitivity (r = 0.353, *p* < 0.05). Beneficial *sisu* was also positively correlated with self-reported risk-taking, as measured by GRiPS and the Stimulating-Instrumental Risk Inventory (SIRI).

*Economic worry* and *difficulty affording necessary expenses* were weakly but negatively associated with HAT points (r = −0.274, *p* < 0.1; a non-significant trend, and r = −0.318, *p* < 0.05). Economic worry was also linked to reinforcement sensitivity following rewards (r = 0.342, *p* < 0.05; r = 0.309, *p* < 0.05) and punishment (r = −0.257, *p* < 0.1; r = −0.264, *p* < 0.1, non-significant trends). A non-significant trend was observed between penalty sum and financial difficulty (r = 0.281, *p* < 0.1).

Regarding BART (Balloon Analogue Risk Task) variables, only a few significant associations or trends emerged. There was a positive correlation between a bolder HAT aiming point and the number of BART pumps (r = 0.368, *p* < 0.05). Additional BART associations can be found in Table 4.

### 6.4. Regression Analyses

To assess the predictive power of HAT and BART measures for self-reported risk-taking tendencies (GRiPS and SIRI scores), stepwise regression analyses were conducted (Table 5 and Table 6). Behavioural variables were included based on two criteria: 1. Strong correlations with self-reported measures. 2. No strong intercorrelations were found between HAT and BART variables. Reinforcement sensitivity was combined into a single variable by normalising the separate reward and punishment sensitivity measures.

The analyses demonstrated that BART variables had little predictive power for self-reported risk-taking tendencies. In contrast, GRiPS risk-taking scores were significantly associated with HAT reinforcement sensitivity (β = 0.466, *p* = 0.002) and were moderately predicted by the complete model (adjusted R^2^ = 0.181). SIRI risk-taking scores showed a marginal association with reinforcement sensitivity (β = 0.316, *p* = 0.046) and were not well-predicted by the complete model (adjusted R^2^ = 0.054).

## 7. Summary of Results

### 7.1. Study 1

This study confirmed that participants responded to both higher penalty levels and greater deviations of the “gun” in the HAT by taking fewer risks. These findings support the validity of HAT as a tool for measuring risk-taking preferences.

### 7.2. Studies 2, 3, and 4

Across these studies, several associations were observed between HAT variables and personality traits, though the findings were not entirely consistent. Extraversion was positively correlated with some risk-taking variables in HAT in Studies 2 and 3 but not in Study 4. In Study 2, lower family SES was associated with higher reinforcement sensitivity in HAT. Extraversion was also positively correlated with SES and self-reported real-life risk behaviour. Study 3 found a negative association between depressive symptoms (BDI) and HAT performance (points). In Study 4, economic worry and difficulty affording necessary expenses were associated with poor HAT performance and heightened reward and punishment sensitivity. Study 3 found a strong association between agreeableness and punishment sensitivity in HAT. However, this association was not found in Studies 2 and 4. A notable difference in Study 4 was the use of constant deviation in HAT, whereas Studies 2 and 3 employed shifting deviations, which might have influenced participant behaviour. Additionally, Study 3 had a smaller sample size, which may have limited the reliability of its findings.

### 7.3. Self-Reported Risk-Taking (Study 4)

Extraversion was associated with risk-taking measured by GRiPS, but not by the SIRI scale. Honesty/humility was negatively correlated with both GRiPS and SIRI scores. HAT reinforcement sensitivity variables (aiming shifts after rewards and penalties) were associated with self-reported risk-taking tendencies measured by GRiPS and SIRI in regression analyses, while BART variables showed no predictive power. Beneficial sisu was associated with reinforcement sensitivity and self-reported risk-taking variables (GRiPS and SIRI).

### 7.4. Summary

The HAT demonstrated utility in assessing risk-taking behaviour and its associations with personality traits and socioeconomic factors, particularly through reinforcement sensitivity measures. However, inconsistencies in findings across studies highlight the importance of experimental conditions, sample sizes, and task designs in interpreting results.

## 8. Discussion

Our results indicate that several risk-taking variables in the HAT were associated with various psychological traits and external factors, including socioeconomic status (SES) and risk-taking propensity, across multiple study samples. Extraversion was linked to risk-taking tendencies in HAT in two out of three samples. Additionally, reinforcement sensitivity (indicated by stronger shifts in aiming after reward and punishment) was associated with several external variables, such as self-reported risk-taking tendencies (measured by GRiPS and SIRI), family SES, economic worry, and the difficulty of affording necessary expenses.

HAT demonstrated strong external construct validity, as it showed significant associations with other relevant variables. In comparison to the Balloon Analogue Risk Task (BART), in our sample, HAT exhibited better convergent validity based on its correlations with multiple self-reported questionnaires. A notable advantage of HAT is the more detailed insights into decision-making processes it provides in comparison to other behavioural risk-taking measures. Each aiming point can be individually evaluated, allowing for the calculation of separate statistics, including the average shift in aiming point, the shift in aiming point after penalty, and the average deviation from the optimal aiming point in response to reward and punishment. Additionally, the fine-grained information of HAT provides an opportunity to study brain activity and compare it between individuals and trials in which punishment leads to less or more risk-taking. In comparison to tasks in which the risk accumulates over actions so that the likelihood of ‘popping’ the balloon will increase towards 100% at some point, in the HAT, each individual click of the button indicates a separate risk decision, therefore providing a good time resolution to study the behavioural and neural reactions to the consequences of each action.

Previous literature does not fully align with our current findings. de Vries et al. (2009) reported that honesty/humility, emotionality, conscientiousness, and, to a lesser degree, agreeableness were negatively correlated with self-reported risk-taking (IPIP scale), while extraversion and openness showed a positive correlation with risk-taking. Similarly, Nicholson et al. (2005) found that extraversion and openness were positively correlated with self-reported risk-taking (DOSPERT scale), whereas neuroticism, agreeableness, and conscientiousness were negatively associated with risk-taking. However, other studies have failed to replicate the association between extraversion and risk-taking (Weller & Tikir, 2011; Weller & Thulin, 2012), suggesting that the relationship between personality traits and risk-taking tendencies remains unclear.

In a large sample with multiple different risk-taking measures, openness and extraversion were found to be associated with risk-taking propensity (Frey et al., 2017). Lejuez et al. (2002), in turn, found that risk-taking in the Balloon Analogue Risk Task (BART) correlated with self-reported real-life risk behaviours and personality traits associated with risk-taking, such as sensation seeking, impulsivity, and low behavioural constraint. On the other hand, Brailovskaia et al. (2018) found that BART did not predict real-life risk-taking, further highlighting the inconsistencies in the predictive power of behavioural tasks in relation to actual risk-taking behaviour.

There is no previous research on the potential relationship between risk-taking and beneficial versus harmful sisu, making this study the first to explore this connection. Surprisingly, we found that beneficial sisu was positively associated with reinforcement sensitivity, as well as self-reported risk-taking measures (GRiPS and SIRI). This finding is unexpected, as beneficial sisu is typically regarded as a positive trait linked to better mental health, better well-being, and lower levels of stress.

In terms of socioeconomic status (SES), lower family SES is generally thought to be associated with higher risk-taking behaviour during adolescence (Kipping et al., 2015). Our findings align with this perspective, as we observed that lower family SES was associated with higher reinforcement sensitivity in the HAT among adolescents.

What do our findings regarding “reinforcement sensitivity” in the HAT suggest? According to Gray’s (1982) Reinforcement Sensitivity Theory, the Behavioral Inhibition System (BIS) is associated with punishment sensitivity, while the Behavioral Activation System (BAS) is linked to Reward Responsiveness. BAS can be defined as “one’s ability to experience pleasure in the anticipation and presence of reward-related stimuli” (Taubitz et al., 2015). Based on the results from Studies 2, 3, and 4, which show shifts in aiming after reward and punishment correlate with each other, we interpret these as reflecting higher reinforcement sensitivity—specifically, stronger responses to both reward and punishment.

As mentioned in the introduction, there are many ways in which HAT behaviour, especially aiming shifts after rewards and penalties, could be interpreted, and calling it ‘reinforcement sensitivity’ is only a tentative label for it. The behaviours could as well represent mechanisms of higher cognition and information processing, or, perhaps most likely, some combination of different levels of processing.

Previous research has linked BAS and its subscales to performance on the Iowa Gambling Task (Demaree et al., 2008; Franken & Muris, 2005; Herman et al., 2018; Suhr & Tsanadis, 2007), though some studies have not found this association (Studer et al., 2013). Our findings are consistent with the notion that greater reinforcement sensitivity is related to more pronounced reactions to reward and punishment in decision-making tasks.

The associations between extraversion and HAT observed in Studies 2 and 3 were not replicated in Study 4. However, it is important to note that the Study 4 sample had some notable differences. In Study 4, the HAT had a constant variance, which was not altered during the experiment, making the task more predictable. Additionally, the starting point for aiming was shifted from the centre to the bottom-left corner of the screen, which may have influenced participants’ behaviour.

In Study 2, conducted among adolescent males, the Consideration for Future Consequences (CFCs) scale was positively associated with openness and conscientiousness and negatively with self-reported real-life risk behaviours, such as substance use and gambling. Cosenza et al. (2019) also found that CFC was linked to real-life gambling problems. In contrast, in our study, CFC was not associated with any of the HAT variables, highlighting a potential difference in the relationship between future-oriented thinking and risk-taking behaviours across different experimental tasks.

Risk-taking tendency also has a partially biological background, and thus it is not surprising that risk-taking tendency-like traits, like novelty seeking, have been associated with higher testosterone levels among men (Määttänen et al., 2013). Risk-taking tendency among animals in different forms has been a popular topic of research, but it has been found that it is influenced by the given species and their evolutionary history, and contextual factors (for a review, see Houston & Rosenström, 2024). Utilising self-reported or self-report-based evaluations (“you will receive the reward at time X”) in studying risk preferences and behaviours has its benefits, but it seems to hamper the comparisons between humans and non-human animal risk-taking tendency. Non-human animals and humans seem to behave in a more similar manner when studied with similar behavioural experimental setups, rather than utilising self-report-based methods for humans (for multiple examples, see Houston & Rosenström, 2024). It has been argued that more human studies should involve behavioural tasks to allow comparability with non-human animal behaviours (Määttänen et al., 2021).

In addition, future studies should include possible modelling of the behaviour towards immediate reward and punishment. For instance, it is possible that there would be longer-term differences between individuals in terms of their responses to reward and punishment, which may not be clear from the currently used numbers. The HAT variables could and should be contrasted with multiple risk-taking variables to better understand their relationship. For instance, which HAT variables are highly loaded onto the proposed general risk-taking propensity factor, or “R”? Also, the temporal stability of HAT variables should be explored, as it has been found that behavioural risk-propensity measures have lower temporal stability than self-report-based risk-taking propensity measures.

In the future, HAT could and should be studied in diverse populations, including clinical and prison inmate populations. Besides self-reported scales, objective measures of extreme risk-taking would be beneficial. Compared to other behavioural tests, HAT includes the possibility of analysing the immediate impacts of positive and negative outcomes on different types of individuals, including those suffering from addictions. More age groups should be studied with HAT. Longitudinal data on HAT, including test–retest reliability and learning from one session to another, should be examined. Converting HAT to an online version in the future would be useful for gaining access to larger samples of participants.

Future research is needed to confirm the results of this study, and the HAT algorithm (code) is publicly available alongside this study article to facilitate further investigation (see File S1). We hope that the HAT will be actively utilised and validated by other researchers and implemented in practical applications in the future.

A limitation of this study is the inconsistency of some findings across the smaller study samples.

By exploring the complexities of risk-taking propensity, this research aims to lay the groundwork for a more comprehensive understanding of human decision-making behaviour through the novel HAT. Ultimately, our goal is to contribute to both theoretical models and practical applications in fields where effective risk management and decision-making are crucial. HAT presents a promising addition to the research of risk-taking, as it allows examining risk-taking tendencies and reinforcement sensitivity simultaneously. Future studies should incorporate real-life risk-taking outcomes to further validate the effectiveness of HAT in predicting and understanding risk-related behaviours.

Finally, we note that some of the present findings, particularly those related to reinforcement sensitivity, should be interpreted with caution due to the relatively small and heterogeneous samples involved. Rather than providing definitive evidence, these results offer preliminary indications that call for replication in larger and more diverse populations. The construct of reinforcement sensitivity as applied in the HAT framework also remains tentative. Our interpretation reflects one possible account among several, and alternative mechanisms—such as attentional fluctuations, learning effects, or variations in task engagement—may equally contribute to the observed patterns. Future research should aim to explicitly model and control for these factors to clarify their relative roles.

From a theoretical and applied perspective, further work is needed to establish the practical relevance of HAT-derived measures. While the present findings suggest promising external validity, future studies should examine how HAT indices of reinforcement sensitivity and risk-taking relate to real-world behaviours, such as health, financial, and educational decisions. Moreover, given the inherent limitations of self-report measures, subsequent research would benefit from integrating behavioural and physiological indicators to provide a more comprehensive understanding of risk-taking processes. Refining both the conceptual and methodological foundations of HAT will be essential for advancing its utility as a robust tool for studying individual differences in reinforcement and decision-making.

## Figures and Tables

**Table 1:** Optimal aiming points for different standard deviations (i.e., uncertainty of the shot from the aiming location; standard deviations from the aiming point) and penalties (i.e., what is the most negative outcome of the shot in the current table).

	Horizontal Standard Deviation		
Penalty	1.5	1.8	2.1
−100	7.10	6.44	5.81
−50	7.46	6.89	6.34
0	8.77	8.53	8.34
Mean	7.77	7.29	6.83

**Table 2:** Mean aim locations and standard deviations separately for both groups, gun accuracy (horizontal dispersion), and the level of penalty.

	Mean Aim Locations (Standard Deviation)				
	Group 1		Group 2		Combined Groups
Level of Penalty	1.8	1.5	1.8	2.1	1.8
−100	6.34 (1.09)	6.73 (0.87)	6.79 (0.75)	6.23 (0.75)	6.56 (0.96)
−50	6.88 (0.94)	7.19 (0.77)	7.22 (0.61)	6.63 (0.55)	7.04 (0.80)
0	8.63 (0.58)	8.74 (0.61)	8.35 (0.70)	8.03 (0.62)	8.49 (0.65)
Levels combined	7.28 (0.71)	7.55 (0.53)	7.45 (0.53)	6.96 (0.45)	7.37 (0.63)

**Table 3:** Pearson correlation coefficients between HAT-derived variables and other variables in Study 2.

	1.	2.	3.	4.	5.	6.	7.	8.	9.	10.	11.	12.	13.
1. HAT points	1												
2. HAT aim from optimum	**−0.247 ^**	1											
3. HAT penalty sum	−0.543 ***	0.716 ***	1										
4. HAT penalty count	−0.557 **	0.710 ***	0.991 ***	1									
5. HAT aiming shift after penalty	0.017	0.184	0.105	0.107	1								
6. HAT aiming shift after reward	−0.285 *	0.136	0.265 *	0.275 *	−0.655 ***	1							
7. Considerations of Future Consequences	−0.014	−0.063	−0.033	−0.044	0.037	−0.002	1						
8. Neuroticism	−0.046	−0.107	−0.129	−0.137	0.011	−0.099	−0.218	1					
9. Extraversion	−0.142	**0.261 ^**	**0.262 ^**	0.293 *	−0.010	0.254	−0.025	−0.346 **	1				
10. Openness	0.068	−0.033	−0.277 *	−0.248	0.108	−0.105	0.321 *	0.012	0.008	1			
11. Agreeableness	−0.046	−0.096	−0.074	−0.079	−0.054	0.034	−0.028	−0.066	−0.178	0.200	1		
12. Conscientiousness	0.134	0.140	−0.015	−0.014	0.151	−0.100	0.474 ***	−0.381 **	0.194	0.402 **	0.141	1	
13. Family Socioeconomic Status (log SES)	0.048	−0.075	−0.104	−0.086	0.363 *	−0.348 *	−0.133	0.199	**−0.274 ^**	0.107	−0.044	−0.006	1
14. Risk-taking behaviour and substances (log)	−0.103	0.299 *	0.120	0.121	0.169	−0.046	−0.259 *	−0.063	0.276 *	0.044	−0.205	−0.134	0.060

*p*-values: *** = *p* < 0.001; ** = 0.01; * = 0.05; ^ = 0.1. Note: aiming shift after penalty is typically always negative, which means that a negative correlation can be interpreted as a positive correlation. On the other hand, aiming shift after reward is typically positive, which means that a positive correlation means more aiming shift to a riskier direction.

**Table 4:** Pearson correlation coefficients between HAT-derived variables and other variables in Study 4.

	1.	2.	3.	4.	5.	6.	7.	8.	9.	10.	11.	12.	13.	14.	15.	16.	17.	18.	19.
1. HAT Points	1																		
2. HAT aim from optimum	−0.485 **	1																	
3. HAT penalty sum	−0.963 ***	0.699 ***	1																
4. HAT penalty count	−0.924 ***	0.742 ***	0.979 ***	1															
5. HAT aiming shift after penalty	0.362 *	0.171	**−0.245 ^**	−0.177	1														
6. HAT aiming shift after reward	−0.482 **	0.079	0.421 **	0.378 **	−0.771 ***	1													
7. BART number of pumps	−0.030	0.368 *	0.141	0.162	0.101	0.004	1												
8. BART earnings	0.043	0.248	0.041	0.039	0.184	−0.169	0.870 ***	1											
9. BART number popped	−0.044	0.407 **	0.166	0.201	0.086	0.094	0.884 ***	0.567 ***	1										
10. GRiPS (risk questionnaire)	−0.319 *	0.089	0.301 *	0.254	−0.348 *	0.397 **	0.136	0.095	0.11	1									
11. SIRI (risk questionnaire)	−0.220	0.123	0.231	0.217	−0.218	0.370 *	0.216	0.141	**0.251 ^**	0.789 ***	1								
12. Openness	0.211	−0.200	−0.218	−0.200	−0.013	−0.004	0	−0.048	0.026	0.067	0.014	1							
13. Extraversion	−0.104	0.047	0.102	0.112	−0.236	0.125	−0.070	−0.070	−0.112	0.416 **	0.183	0.183	1						
14. Emotionality	0.027	−0.07	−0.041	−0.084	−0.085	0.115	−0.243	−0.240	−0.159	**−0.283 ^**	−0.170	−0.069	−0.355 *	1					
15. Honesty-Humility	0.096	−0.076	−0.112	−0.085	0.074	−0.090	0	−0.032	0.028	−0.464 **	−0.441 **	0.285	−0.232	0.150	1				
16. Agreeableness	−0.044	**0.283 ^**	0.127	0.171	−0.121	0.051	0.372 *	0.339 *	0.324 *	0.062	0.229	−0.173	−0.077	−0.047	0.007	1			
17. Conscientiousness	−0.200	0.209	0.224	0.187	**−0.246 ^**	0.153	−0.112	−0.119	−0.079	0.102	0.018	0.242	0.207	−0.296 *	0.179	0.012	1		
18. Harmful Sisu	−0.075	−0.063	0.051	0.028	0.070	−0.029	0.016	0.030	0.047	0.123	0.150	0.086	−0.222	−0.034	−0.186	−0.158	−0.116	1	
19. Beneficial Sisu	−0.193	−0.08	0.139	0.133	−0.339 *	0.353 *	−0.028	−0.059	−0.055	0.647 **	0.438 **	0.365 *	0.587 **	−0.410 **	−0.150	−0.109	0.335 *	0.018	1
20. Mental Toughness	0.010	−0.035	−0.017	−0.008	−0.210	0.060	0.167	0.095	0.156	0.418 **	0.250	0.234	0.527 ***	−0.525 ***	−0.086	0.066	0.378 **	−0.469 **	0.657 ***

*p*-values: *** = *p* < 0.001; ** = 0.01; * = 0.05; ^ = 0.1. Note: aiming shift after penalty is typically always negative, which means that a negative correlation can be interpreted as a positive correlation. On the other hand, aiming shift after reward is typically positive, which means that a positive correlation means more aiming shift to a riskier direction.

**Table 5:** Stepwise regression analyses when predicting self-reported GRiPS risk-taking tendency with HAT and BART-variables.

	Model 1.			Model 2.			Model 3		
	β	t-Value	Sig.	β	t-Value	Sig.	β	t-Value	Sig.
HAT reinforcement sensitivity	0.319	2.334	0.024	0.347	2.568	0.014	0.466	3.308	0.002
HAT aim from optimum				0.357	2.015	0.050	0.261	1.447	0.155
HAT penalty sum				0.184	1.033	0.307	−0.003	−0.016	0.987
BART earnings							0.287	1.907	0.063
BART popped							−0.264	−1.775	0.083
R-squared	0.102			0.177			0.264		
Adjusted R-squared	0.083			0.123			0.181		
R-squared change	0.102			0.075			0.087		
Sig. of the R-squared change	0.024			0.134			0.085		

**Table 6:** Stepwise regression analyses when predicting self-reported SIRI risk-taking tendency with HAT and BART-variables.

	Model 1.			Model 2.			Model 3		
	β	t-Value	Sig.	β	t-Value	Sig.	β	t-Value	Sig.
HAT reinforcement sensitivity	0.211	1.496	0.141	0.235	1.663	0.103	0.316	2.086	0.043
HAT aim from optimum				0.307	1.659	0.104	0.212	1.094	0.280
HAT penalty sum				0.156	0.840	0.405	0.038	0.188	0.852
BART earnings							0.259	1.603	0.116
BART popped							−0.080	−0.500	0.619
R-squared	0.045			0.100			0.150		
Adjusted R-squared	0.025			0.042			0.054		
R-squared change	0.045			0.056			0.050		
Sig. of the R-squared change	0.141			0.250			0.287		

## Data Availability

The data cannot be shared due to privacy concerns.

## Supplementary Materials

The following supporting information can be downloaded at the following link: https://www.mdpi.com/article/10.3390/bs15111597/s1, File S1: Includes the Python-code for the risk-taking game, HAT. File S2: Includes the Supplementary Tables S1–S3.
