# Supplementary material for: Development and Validation of a New Risk-Taking Game: Helsinki Aiming Task (HAT)
Source: Behav Sci (Basel). 2025 Nov 20;15(11):1597. doi: 10.3390/bs15111597 (PMC12649229; doi:10.3390/bs15111597)
Supplement: Supplementary file 1 [file behavsci-15-01597-s001.zip › behavsci-3769888-supplementary/behavsci-3769888-File S2.pdf]

Supplementary Table S1. Descriptives for Study 2. A positive value for "HAT aim from optimum" indicates a riskier aiming point relative to the optimum. A negative value for "HAT aiming shift after punishment" signifies a shift towards the low-risk end of the spectrum, while a positive value for "HAT aiming shift after reward" reflects a shift towards the high-risk end following a successful outcome in the previous "shot."

	Mean	SD	Min	Max	N
HAT points	2012.59	781.16	232.00	3072	61
HAT aim from optimum	0.45	0.68	-1.17	2.111	60
HAT penalty sum	1715.00	1155.43	0	7250.00	60
HAT penalty count	23.35	15.88	0.00	96	60
HAT aiming shift after punishment	-0.888	0.711	-3.510	0	60
HAT aiming shift after reward	0.045	0.045	-0.027	0.164	60

Supplementary Table S2. Descriptives for Study 3. A positive value for "HAT aim from optimum" indicates a riskier aiming point relative to the optimum. A negative value for "HAT aiming shift after punishment" signifies a shift towards the low-risk end of the spectrum, while a positive value for "HAT aiming shift after reward" reflects a shift towards the high-risk end following a successful outcome in the previous "shot."

	Mean	SD	Min	Max	N
HAT points	1967.56	874.46	378	3194	18
HAT aim from optimum	0.49	0.84	-1.66	1.63	18
HAT penalty sum	1750	927	600	3600	18
HAT penalty count	24.11	13.23	7	49	18
HAT aiming shift after punishment	-0.62	0.61	-2.04	0.00	18
HAT aiming shift after reward	0.025	0.037	-0.019	0.136	18

Supplementary Table S3. Descriptives for Study 4. A positive value for "HAT aim from optimum" indicates a riskier aiming point relative to the optimum. A negative value for "HAT aiming shift after punishment" signifies a shift towards the low-risk end of the spectrum, while a positive value for "HAT aiming shift after reward" reflects a shift towards the high-risk end following a successful outcome in the previous "shot."

	Mean	SD	N
HAT points	2223.57	750.09	47
HAT aim from optimum	0.51	0.51	47
HAT penalty sum	1522.34	803.31	47
HAT penalty count	21.06	11.30	47
HAT aiming shift after punishment	-0.89	0.64	47
HAT aiming shift after reward	0.03	0.04	47
BART pumps	27.42	12.13	47
BART earnings	1.02	0.38	47
BART popped	5.19	2.59	47
GRiPS	2.66	0.89	47
SIRI	2.18	0.43	47
Openness	4.20	0.62	47
Extroversion	4.12	0.76	47
Emotionality	2.98	0.65	47
Honesty-Humility	3.83	0.75	47
Agreeableness	2.87	0.73	47
Conscientiousness	3.24	0.68	47
Harmful Sisu	3.51	1.17	47
Beneficial Sisu	4.85	1.06	47
Mental Toughness	33.57	6.67	47
